# A Case of Ovariotomy

**Published:** 1861-01

**Authors:** Henry Miller

**Affiliations:** Formerly Professor of Midwifery in the University of Louisville


ART. V.—*A Case of Ovariotomy.* By HENRY MILLER, M.D., formerly Professor of Midwifery in the University of Louisville.

IN a paper read before the College of Physicians and Surgeons of Louisville, and published in the *American Journal of the Medical Sciences* for April, 1859, I gave an account of two cases of cystic ovary successfully treated by excision, and attempted to justify the operation of ovariotomy under certain circumstances. Having recently had another successful case, I now propose to present a brief notice of it.

Mrs. T., forty-eight years of age, consulted me on account of a multilocular degeneration of the left ovary, which attracted her attention as a tumor upwards of a year since, but she had complained of pain in the left iliac region and limb of the same side for several years previously. The tumor, when she came under my observation, had grown to a large size, and invaded all the regions of the abdomen, not excepting the hypochondriac, and almost diagnosticated itself upon the surface by the prominence of the cysts and the depth of the sulci between them.

I operated upon her on the eighteenth of October, in the usual way, by making an incision through the linea alba from the umbilicus to within an inch of the pubes. This brought to view the main cyst, which was punctured, and its fluid contents received in a vessel. The superior cysts were then pressed down by an assistant, and held opposite the abdominal opening, and their fluid collected. Several smaller cysts were opened and their contents allowed to escape at random. After all this depletion the solid textures of the tumor could barely be brought through the incision. The collected fluid measured twenty pints, and the solid remnant of the growth, which was extracted, weighed eight pounds. Allowing that only two pounds of fluid were not collected, which was the lowest estimate made by those who were present, the tumor must have weighed thirty pounds before it was molested.

It was my purpose to have secured the pedicle with the clamp, proposed by Mr. Spencer Wells, but when I observed how large was the main artery supplying it, and how deeply its tissues were congested, I concluded that it would be safer to use a strong ligature of flax, and to secure the artery by a special ligature. The clamp was then applied, with the view of retaining the stump exterior to the cavity of the abdomen, and in this respect only did I depart from the method pursued in my two previous cases. If, when the pedicle is left in the abdominal cavity, the suppuration and sloughing are equal to what I observed on the outside, and there is no reason to suppose that there is any difference, then I am disposed to think that it would be always better to proceed as I did in the present case, for there must be danger when the pedicle is left within the body, not only of peritonitis, but of purulent absorption. The sloughing was not completed until the twentieth day after the operation, when the remnant of the stump was not larger than a cherry, and was retracted within a deep depression, looking like the umbilicus of a newly born child.

The external incision was closed with the interrupted suture of annealed iron wire, and the union was firm and complete in seven or eight days. This was the first time that I had used metallic sutures, and I was not very favorably impressed by them. There was fully as much suppuration along their track as I have been accustomed to see, when sutures of silk or flax were employed, and there was, moreover, a small abscess formed in connection with the suture, next to the umbilicus. This might have been, however, only a coincidence, and I do not feel authorized to decry what others have so much lauded, judging from only a single observation.

No threatening symptoms followed the operation. There was no shock, no tenderness or fullness of the abdomen, nor any fever. Her appetite continued good enough throughout. Her pulse ranged from eighty to ninety in the minute; she slept well, and complained only of a little backache and some pain in the left leg, which readily yielded to a liniment of chloroform. During the fourth week she sat up several hours every day, and walked about the room, and just one month and a day after the operation she returned home, making the journey in one day, partly by rail, and partly by riding in a carriage thirty miles, without inconvenience of any kind.

It is hardly necessary to add that Mrs. T. was placed completely under the influence of chloroform, preparatory to the operation. To the immunity from suffering and consequent shock, thus purchased, I ascribe, in no small degree, the fortunate issue of this, as well as my two previous cases. No consciousness of pain was evinced by her except from the sewing up of the external wound, and she afterward declared that she felt no pain from anything else that was done.

